# ER22/23EK polymorphisms of the glucocorticoid receptor are not associated with the response of alcohol‐associated hepatitis patients to prednisolone treatment

**DOI:** 10.14814/phy2.71163

**Published:** 2026-11-08

**Authors:** Navakanth Raju Ramayanam, Rajesh Nanda Amarnath, Sarad Pawar Naik buke, Vijayakumar Thangavelmahalingam, Abdullateef Isiaka Alagbonsi, Hope Onohuean

**Affiliations:** ^1^ Department of Pharmacy Practice, Faculty of Pharmacy Dr. M.G.R. Educational and Research Institute Chennai Tamil Nadu India; ^2^ Department of Medical Gastroenterology SRM Medical College Hospital and Research Center Kattankulathur, Kanchipuram Tamil Nadu India; ^3^ Department of Pharmacy, Faculty of Health Sciences Victoria University Kampala Uganda; ^4^ Department of Pharmacy Practice, SRM College of Pharmacy SRM Institute of Science and Technology Kattankulathur, Kanchipuram Tamil Nadu India; ^5^ Department of Physiology, School of Medicine and Pharmacy, College of Medicine and Health Sciences University of Rwanda Huye Southern Province Republic of Rwanda; ^6^ Biomolecules, Metagenomics, Endocrine and Tropical Disease Research Group (BMETDREG) Kampala International University, Western Campus Bushenyi Uganda; ^7^ Biochemistry Programme, College of Agriculture, Engineering and Science Bowen University Iwo Nigeria

**Keywords:** alcohol‐associated hepatitis, ER22/23EK, glucocorticoid receptor, Lille score, pharmacogenetics, prednisolone, tumor necrosis factor‐alpha

## Abstract

The effects of prednisolone on biochemical parameters in patients with alcohol‐associated hepatitis (AH) have been reported. However, data on the pharmacogenetic determinants of prednisolone response in these patients remain limited. Because the ER22/23EK polymorphism of the glucocorticoid receptor gene (NR3C1) has been linked with relative glucocorticoid resistance in other settings, this study investigated its prevalence and influence among prednisolone‐responsive and non‐responsive AH patients. A total of 134 patients with AH were enrolled in this prospective study. Biochemical parameters diagnostic of liver function, messenger ribonucleic acid (mRNA) expression of tumor necrosis factor‐alpha (TNF‐α), and ER22/23EK (rs6189 and rs6190) genotypes were assessed. Prednisolone response was defined using the Lille score after 7 days of therapy. Beyond classical biochemical parameters of liver function and the Lille score, ER22/23EK genotype polymorphisms of the glucocorticoid receptor were not associated with patients' response to prednisolone treatment in this cohort. TNF‐α mRNA expression was higher in AH patients who responded to prednisolone than in those who did not respond to treatment. TNF‐α mRNA was higher at day 7 among prednisolone responders than non‐responders. More importantly, in this cohort, prednisolone response among AH patients was not statistically associated with the ER22/23EK genotype. These findings should be interpreted cautiously because additional glucocorticoid receptor targets, 11β‐hydroxysteroid dehydrogenase expression, and functional GR assays were not assessed.

## 1 INTRODUCTION

Alcohol‐associated liver disease (ALD), within the broader steatotic liver disease (SLD) nomenclature, remains a major cause of cirrhosis‐related morbidity and mortality worldwide (Gao & Bataller, 2011; Maras et al., 2018). Contemporary terminology increasingly avoids the term “alcoholic” and instead uses alcohol‐associated or alcohol‐related liver disease to reduce stigma and align with current SLD nomenclature recommendations (Rinella et al., 2023, 2024). Alcohol‐associated hepatitis (AH) is a severe inflammatory presentation of ALD that may progress to acute‐on‐chronic liver failure, infection, and early mortality (Sarin & Sharma, 2020). Clinically, AH is characterized by jaundice in the context of recent heavy alcohol intake, an AST/ALT ratio typically greater than 1.5, and exclusion of competing liver diseases. Therefore, identifying predictors of corticosteroid response is clinically important because prednisolone remains a major therapeutic option for severe AH, but a substantial proportion of patients do not respond (Sarin & Sharma, 2020).

AH is characterized by recent onset or worsening of jaundice in individuals with persistent harmful alcohol consumption up to at least 6 weeks before clinical presentation. It is also associated with elevated liver enzymes, with an aspartate aminotransferase (AST) to alanine aminotransferase (ALT) ratio of >1.5:1 and absolute aminotransferase values that usually do not surpass 500 IU/L, after exclusion of alternative liver disorders (Gan et al., 2025). Gender, drinking pattern, genetic predisposition, and concurrent liver conditions are recognized risk factors that can guide the onset and advancement of liver disease (Jamalinia et al., 2024). The pathogenesis of AH is multifactorial and includes steatosis, increased intestinal permeability, oxidative stress, toxic ethanol metabolites, innate immune activation, and cytokine generation (Onohuean et al., 2025; Wang et al., 2024).

Early detection and targeted treatment are crucial in AH, since severe alcohol‐associated hepatitis (SAH) can culminate in critical liver failure, high infection risk, and significant mortality. In the absence of highly effective therapeutics, corticosteroids have been suggested as a beneficial treatment for selected patients with SAH (Saberi et al., 2016). Corticosteroids act through cytoplasmic glucocorticoid receptors (GRs) to mediate their biological actions (Ramamoorthy & Cidlowski, 2016). By altering cytokine stability and transcriptional activity, corticosteroids suppress pro‐inflammatory cytokines such as TNF‐α and promote anti‐inflammatory mediators such as interleukin (IL)‐10 (Barnes, 2006). Prednisolone has therefore been recommended for selected individuals with severe AH for several decades (Philips et al., 2019).

The glucocorticoid effects are mediated through genomic mechanisms, which have a slower onset but longer duration, and non‐genomic mechanisms, which have a rapid onset but shorter duration. Some of the affected genes have been reportedly associated with nephrotic syndromes (Adepoju et al., 2025; Ajayi et al., 2025). In the genomic pathway, ligand‐bound GR translocates to the nucleus and regulates gene transcription through glucocorticoid response elements and interaction with inflammatory transcription factors such as NF‐κB and AP‐1 (Schijvens et al., 2019). Thus, the term “modulation of gene transcription” is more accurate than “genetic modification” because prednisolone does not edit or permanently modify patient DNA (Evans & McLeod, 2003; Weinshilboum & Wang, 2004). Several published studies in severe AH have evaluated prednisolone response using clinical endpoints and Lille‐score‐based non‐response (Forrest et al., 2018; Sabatose et al., 2025); however, pharmacogenetic data in AH remain sparse.

Variation in the NR3C1 gene coding for GRs can affect glucocorticoid sensitivity (DeRijk et al., 2002; Dobson et al., 2001; Huizenga et al., 1998). ER22/23EK is a relatively uncommon linked polymorphism in codons 22 and 23: the codon 22 change is silent, whereas the codon 23 change substitutes lysine for arginine (R23K) (Echwald et al., 2001; Rosmond et al., 2001; van Rossum et al., 2002). The polymorphism has been associated with relative glucocorticoid resistance, altered GR translational isoform expression, lower inflammatory markers, and favorable metabolic phenotypes in non‐AH populations (van Rossum et al., 2002, 2004). Its relevance to AH is therefore biologically plausible but uncertain: prednisolone acts through GR‐mediated transcriptional regulation, while AH outcomes are also strongly driven by liver dysfunction, infection risk, bilirubin kinetics, creatinine, prothrombin time, and ongoing alcohol exposure (Rosmond et al., 2000; van Rossum et al., 2002). Because ER22/23EK is rare and other GR polymorphisms such as N363S and BclI are more frequently studied, the present study should be interpreted as an initial cohort‐level assessment rather than definitive exclusion of all GR pharmacogenetic effects in AH.

A recent pharmacokinetic study compared liver biochemical parameters among Indian patients with AH who either responded or did not respond to prednisolone treatment (Ramayanam et al., 2022). Despite its strength, that study lacked pharmacogenetic data to support precision medicine approaches in AH. Pharmacogenetics has an important role in personalized therapy, as ancestry is reported to play a key role in organ fat accumulation (Hakizimana, Izabayo, et al., 2026) and other phenotypes (Dukuze et al., 2026; Hakizimana & Alagbonsi, 2026; Hosseini et al., 2019; Louvet et al., 2007). Yet, the status of the ER22/23EK polymorphism in prednisolone response among AH patients remains uncertain. To bridge these knowledge gaps, this study was designed to: (i) identify prednisolone responders and non‐responders in patients with AH; (ii) estimate the prevalence and influence of ER22/23EK genotype polymorphism among prednisolone‐responding and non‐responding AH patients; and (iii) assess TNF‐α mRNA expression in responders and non‐responders.

## 2 METHODOLOGY

### 2.1 Patient selection

This research focused on the prospective analysis of individuals diagnosed with early‐stage AH. A total of 340 patients were initially selected for the investigation, of whom 206 were identified as steroid‐ineligible and thus eliminated from the study. Of the remaining 134 patients, 111 were steroid responders (of which 5 later withdrew from the study) while 23 were non‐responders. Subjects enrolled were aged between 18 and 65 years who had previously been identified to have AH using approved criteria, such as ultrasound showing hepatomegaly and varying liver parenchyma, symptoms of portal hypertension, and splenomegaly, subsequently verified by endoscopy along with esophageal or gastric varices. Exclusion criteria for this study included the existence of neoplasia, active infections with hepatitis B or C, hepatorenal syndrome, suspicion of autoimmune liver disease, drug‐induced liver disease, uncontrolled acute infectious conditions, and active gastric ulcers due to corticosteroids.

All enrolled patients met the following requirements to be identified with AH and assigned to the investigative category: early onset of jaundice in an individual with a record of excessive alcohol consumption (60 g/day for men and 40 g/day for women for >6 months), AST/ALT ratio of >1, and bilirubin levels >3 mg/dL. A gastroenterologist examined the subjects' medical data to check for relevant demographic information, laboratory findings, and indications of AH, such as disease activity. After all baseline investigations, prednisolone (40 mg once daily) was prescribed to the patients for 7 days. Prednisolone adherence during the 7‐day assessment period was assessed by direct patient interview and medication review where available; no intentional non‐adherence was documented among patients retained for analysis. Alcohol intake during the 7‐day assessment period was reviewed by clinical interview, but quantitative alcohol‐consumption data were not available for all participants and are therefore acknowledged as a study limitation.

### 2.2 Biochemical serum analysis

Participants underwent a clinical screening process before being selected for the study. Serum samples were screened for hemolysis and lipemia to rule out contamination. Commercial Roche Diagnostics reagents were used to test biochemical markers, including total and direct bilirubin, ALT, AST, alkaline phosphatase (ALP), serum albumin and globulin, albumin/globulin ratio, creatinine, urea, and prothrombin time. Prednisolone 40 mg tablets were used for steroid treatment. The MDF standardized score was utilized to forecast AH severity and need for immediate care, and was calculated as: 4.6 × (Patient PT – Control PT) + TBili. A score of <32 indicated a positive short‐term prognosis. Prednisolone (40 mg, once daily for 7 days) was administered to AH patients with MDF scores greater than 32. The Lille score was used to assess prednisolone responders and non‐responders. Considering total serum bilirubin readings on days 1 and 7, the Lille model score was computed as follows: (exp(*R*))/(1 + exp.(*R*)), where *R* = 3.19 − (0.10 × age) + (0.147 × baseline albumin) + (0.0165 × bilirubin value variation) − (0.206 × creatinine) − (0.0065 × baseline bilirubin) − (0.0096 × prothrombin time). A score of less than 0.45 was identified as a responder, and a score of 0.45 or above as a non‐responder. Patients who responded to therapy were followed up to 28 days.

### 2.3 Genotyping

The GR gene exhibits variations in restriction fragment length during polymerase chain reaction (PCR). Genotyping was performed using genomic DNA isolated from peripheral blood mononuclear cells (PBMCs) collected from enrolled patients. Following the manufacturer's guidelines, genomic DNA was isolated using the Thermo Scientific/Fermentas Genomic DNA Purification Kit (cat. no. K0512; Thermo Fisher Scientific, USA). Using the primer sequences ER22/23EK (rs6189/rs6190), MnlI 5‐GATTCGGAGTTAACTAAAAG‐3 (forward), and 5‐ATCCCAGGTCATTTCCCATC‐3 (reverse), in conjunction with the PCR reaction mixture, DNA segments with polymorphic sites were amplified by PCR. The PCR procedure included a 5‐min denaturation step at 95°C, followed by 30 cycles of denaturation at 94°C for 1 min, annealing at 51°C for 1.5 min, extension at 72°C for 1.5 min, and a final extension at 72°C for 5 min. After digesting 20 μL of the appropriate PCR product (482 bp) at 37°C for 1.5 h with 1.25 units of MnlI restriction enzyme (Thermo Scientific/Fermentas, cat. no. ER1071; Thermo Fisher Scientific), the genotypes of the ER22/23EK polymorphism were ascertained. Ethidium bromide was used to visualize the fragments on a 2% agarose gel. Endonuclease digestion generated fragments of 142 and 163 bp for the wild‐type allele (ER22/23ER genotype); three bands of 142, 163, and 177 bp for the heterozygous allele (ER22/23EK genotype); and 163 and 177 bp fragments for the homozygous allele (EK22/23EK genotype).

### 2.4 RT‐qPCR quantification of TNF‐α mRNA expression

Reverse transcription‐quantitative polymerase chain reaction (RT‐qPCR) was used to determine the cycle threshold (Cq) as a measure of relative TNF‐α mRNA abundance. Following the manufacturer's guidelines, RNA was isolated from PBMCs using a YTA RNA extraction kit (Yekta Tajhiz Azma, Tehran, Iran). Spectrophotometric optical density analysis at 260 and 280 nm was used to quantify RNA quantity and quality. The RevertAid RT Reverse Transcription Kit (cat. no. K1691; Thermo Fisher Scientific) produced cDNA according to the manufacturer's specifications. ABI 7500 real‐time PCR (Applied Biosystems; Thermo Fisher Scientific, Inc.) and SYBR Master Mix (Takara Bio, Inc., Otsu, Japan) were used to quantify TNF‐α expression. Primers were created using the online platforms GenScript and Primer3. The following primer pairs were used: QPCR‐TNFa‐F 5‐TCCTTCAGACACCCTCAACC‐3, QPCR‐TNFa‐R 5‐AGGCCCCAGTTTGAATTCTT‐3, QPCR‐GAPDH‐F 5‐CCTCCAAGGAGTAAGACCCC‐3, and QPCR‐GAPDH‐R 5‐AGGGGTCTACATGGCAACTG‐3. Target gene expression was normalized to GAPDH, which was used as an endogenous control. The amplification protocol involved initial denaturation at 95°C for 30 s, followed by 40 cycles at 95°C for 5 s and 60°C for 34 s. The melting‐curve stage was performed at 90°C for 60 s, 95°C for 15 s, and 60°C for 15 s. Relative expression was calculated using the ΔΔCq approach described by Livak and Schmittgen. For clarity, ΔCq was calculated as Cq(TNF‐α) − Cq(GAPDH), and relative abundance was expressed as normalized fold expression using 2^−ΔΔCq^.

### 2.5 Statistical analysis

SPSS for Windows (Statistical Presentation System Software, SPSS Inc.) version 21.0 was employed to perform statistical analysis. The Shapiro–Wilk test was performed to test normality, and the results were found to be normally distributed. Continuous data are presented as the mean and standard deviation (SD). Unpaired and paired *t*‐test comparisons were performed to compare the two time periods. The chi‐square test was used to compare categorical variables, and Fisher's exact test was used to calculate *p*‐values when appropriate. Statistical significance was set at *p* < 0.05, and exact *p*‐values are reported, where necessary.

## 3 RESULTS

### 3.1 Effects of prednisolone treatment on biochemical parameters

Table 1 shows the biochemical parameters of patients with AH who either did or did not respond to prednisolone medication during the 7‐day treatment period, as a test of liver function. The values on Day 7 were compared with those on Day 1. Our data showed significant decreases (*p* < 0.05) in total serum bilirubin, indirect bilirubin, ALP, serum albumin, globulin, albumin/globulin ratio, serum creatinine, prothrombin time, GGT, and MDF, but significant increases (*p* < 0.05) in ALT, AST, total protein, blood urea, and INR among the prednisolone responders. As the responders were followed up to day 28, their liver‐function parameters were continuously assessed. We observed that the corresponding decreases or increases in their parameters were consistent up to Day 28, demonstrating progressive changes in the parameters in response to prednisolone treatment.

**TABLE 1:** Biochemical parameters of AH patients treated with prednisolone.

Parameters	Days	Mean ± SD (prednisolone responders; *n* = 98)	Mean ± SD (prednisolone non‐responders; *n* = 23)
Age (years)		36.65 ± 5.88	36.57 ± 5.97
Total serum bilirubin (mg/dL)	1	14.30 ± 0.59	20.64 ± 1.02
	7	10.45 ± 0.51^a^	26.52 ± 0.22^a^, ^b^
	14	12.74 ± 3.10^a^	–
	21	11.89 ± 2.34^a^	–
	28	6.59 ± 5.91^a^	–
Direct serum bilirubin (mg/dL)	1	6.70 ± 0.34	6.96 ± 0.78^b^
	7	6.38 ± 0.45	5.24 ± 0.85^a^
	14	6.41 ± 3.94	–
	21	5.90 ± 1.9^a^	–
	28	5.02 ± 3.38^a^	–
Indirect bilirubin (mg/dL)	1	7.77 ± 0.30	8.01 ± 0.69
	7	6.23 ± 0.43^a^	5.25 ± 0.84^a^, ^b^
	14	7.10 ± 3.20^a^	–
	21	6.41 ± 3.94^a^	–
	28	5.58 ± 4.44^a^	–
Alanine aminotransferase (U/L)	1	36.54 ± 1.33	37.13 ± 2.95
	**7**	81.37 ± 5.10^a^	84.17 ± 10.72^a^
	14	34.54 ± 13.10	–
	21	33.62 ± 14.80	–
	28	31.00 ± 42.50	–
Aspartate aminotransferase (U/L)	1	120.14 ± 2.32	121.43 ± 5.04
	**7**	207.22 ± 9.29^a^	195.70 ± 21.55^a^
	14	114.00 ± 26.43	–
	21	109.00 ± 19.43	–
	28	104.80 ± 96.70^a^	–
Alkaline phosphatase (U/L)	1	127.30 ± 1.61	128.13 ± 3.49
	**7**	105.68 ± 1.16^a^	102.52 ± 2.91^a^
	14	116.00 ± 21.40^a^	–
	21	107.00 ± 16.43^a^	–
	28	105.00 ± 12.95^a^	–
Total protein (g/dL)	1	6.48 ± 0.06	6.42 ± 0.14
	7	6.89 ± 0.05^a^	6.97 ± 0.11^a^
	14	5.86 ± 2.9^a^	–
	21	5.10 ± 1.33^a^	–
	28	4.97 ± 0.57^a^	–
Serum albumin (g/dL)	1	2.75 ± 0.06	2.77 ± 0.12
	7	2.49 ± 0.06^a^	2.50 ± 0.14^a^
	14	2.43 ± 0.66^a^	–
	21	1.89 ± 0.30^a^	–
	28	1.20 ± 0.67^a^	–
Serum globulin (g/dL)	1	3.93 ± 0.08	3.84 ± 0.17
	7	3.66 ± 0.08^a^	3.85 ± 0.20
	14	2.87 ± 0.12^a^	–
	21	2.13 ± 0.45^a^	–
	28	1.43 ± 0.26^a^	–
Albumin–globulin ratio	1	0.71 ± 0.02	0.70 ± 0.02
	7	0.64 ± 0.02^a^	0.64 ± 0.01^a^
	14	0.54 ± 0.23^a^	–
	21	0.49 ± 0.03^a^	–
	28	0.45 ± 0.22^a^	–
Serum creatinine (g/dL)	1	0.70 ± 0.02	0.68 ± 0.05
	7	0.57 ± 0.01^a^	0.58 ± 0.02^a^
	14	0.46 ± 0.20^a^	–
	21	0.39 ± 0.16^a^	–
	28	0.37 ± 0.18^a^	–
Blood urea (g/dL)	1	14.14 ± 0.58	13.65 ± 1.30
	7	16.18 ± 0.61^a^	16.35 ± 1.30^a^
	14	11.89 ± 2.34^a^	–
	21	10.67 ± 0.56^a^	–
	28	9.50 ± 0.21^a^	–
International normalized ratio	1	1.71 ± 0.11	1.71 ± 0.24
	7	6.59 ± 0.80^a^	4.71 ± 1.48^a^, ^b^
	14	6.41 ± 0.94^a^	–
	21	5.90 ± 0.90^a^	–
	28	4.28 ± 0.34^a^	–
Prothrombin time (s)	1	15.07 ± 0.70	18.94 ± 0.36^b^
	7	13.79 ± 0.22^a^	16.20 ± 0.17^a^, ^b^
	14	15.37 ± 0.30	–
	21	14.21 ± 0.90	–
	28	13.41 ± 0.17^a^	–
Gamma‐glutamyl transpeptidase	1	318.25 ± 17.64	320.61 ± 40.67
	7	173.70 ± 11.92^a^	174.30 ± 27.53^a^
	14	297.00 ± 13.09^a^	–
	21	221.00 ± 9.49^a^	–
	28	198.00 ± 15.09^a^	–
Maddrey's discriminant function	1	44.09 ± 0.86	49.25 ± 1.67
	7	18.06 ± 0.96^a^	40.72 ± 0.71^a^, ^b^
	14	24.00 ± 2.70^a^	–
	21	15.00 ± 0.35^a^	–
	28	11.55 ± 0.23^a^	–
Lille score	1	–	–
	7	0.11 ± 0.09	0.71 ± 0.15^b^
	14	0.08 ±	–
	21	0.30	–
	28	0.22	–

^a^ *p* < 0.05 versus Day 1 of the corresponding group (prednisolone responder or non‐responder, as applicable).

^b^ *p* < 0.05 versus prednisolone responders of the same corresponding day. Continuous data are reported as mean ± SD.

The parameters in the prednisolone non‐responders showed some similar patterns to the responders' group (*p* < 0.05), except for the observed increase in total serum bilirubin (*p* < 0.05), decrease in direct serum bilirubin (*p* < 0.05), and insignificant change in serum globulin (*p* > 0.05). Between‐group comparisons showed that non‐responders had higher day‐7 total bilirubin, prothrombin time, MDF, and Lille score than responders. Because the Lille model incorporates bilirubin evolution, albumin, creatinine, age, baseline bilirubin, and prothrombin time, the non‐response signal in this cohort appears to have been driven mainly by persistent or worsening bilirubin and coagulation‐related severity rather than by ER22/23EK genotype alone.

### 3.2 Genotypes of the ER22/23EK polymorphism among prednisolone users

It is widely known that endonuclease digestion generates fragments of 142 and 163 bp for the wild‐type allele (ER22/23ER genotype); three bands of 142, 163, and 177 bp for the heterozygous allele (ER22/23EK genotype); and 163 and 177 bp fragments for the homozygous allele (EK22/23EK genotype). Thus, the genotypes of the ER22/23EK (rs6189 and rs6190) polymorphisms among AH patients who either responded or did not respond to prednisolone treatment were recorded. Among the 106 responders, 68 patients had the AA genotype (142 + 163 bp), 31 patients had the AG genotype (142 + 163 + 177 bp), and 7 patients had the GG genotype (163 + 177 bp). Among the 23 non‐responders, 16 patients had the AA genotype, 6 patients had the AG genotype, and 1 patient had the GG genotype (Table 2).

**TABLE 2:** Genotypes of the ER22/23EK polymorphism among prednisolone users.

Genotype	Responders	Non‐responders	Odds ratio	Chi‐square statistics	Degrees of freedom	*p*‐value
AA	68	16	–	0.31	2	0.86
AG	31	6	1.22			
GG	7	1	1.65			

The overall chi‐square test statistic was not significant (*χ* ^2^ = 0.31, *p* = 0.86). Using the ordinary model (without considering dominant or recessive traits), the AG carriers had 1.22 odds while the GG carriers had 1.65 odds of responding to prednisolone treatment, though all the odds ratios were statistically insignificant (*p* > 0.05) (Table 2). To ascertain the insignificance of genotype, we did further analysis that is trait‐sensitive. Under the dominant (AA vs. AG + GG) and the recessive (GG vs. AA + AG) models, genotypes showed no significant association with the response to prednisolone treatment (dominant: OR = 1.28, *p* = 0.81; recessive: OR = 1.56, *p* = 1.00).

### 3.3 Expression of TNF‐α among prednisolone users

The relative abundance of TNF‐α mRNA was lower in AH patients who did not respond to prednisolone treatment (9.14 ± 4.80) than in those who responded to it (25.07 ± 4.82) (*p* = 0.022; Figure 1). This demonstrated that, in this cohort, prednisolone response was associated with higher TNF‐α mRNA expression at day 7.

**FIGURE 1:**
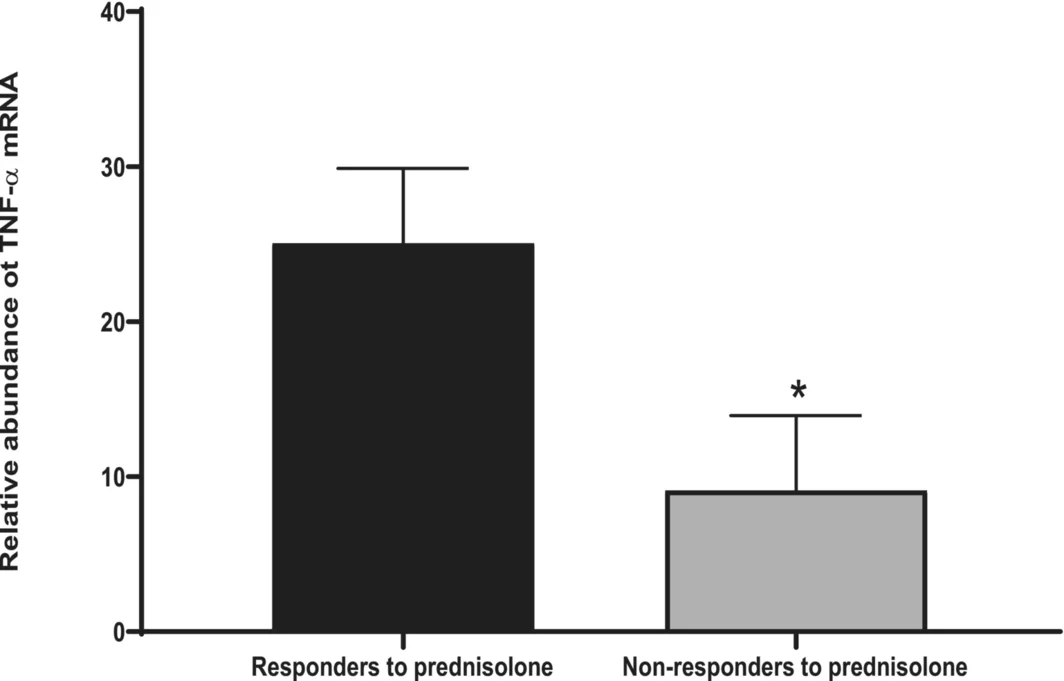
Relative abundance of tumor necrosis factor‐alpha (TNF‐α) messenger ribonucleic acid (mRNA) among alcohol‐associated hepatitis patients treated with prednisolone. Data are presented as mean ± SD. *p = 0.022.

## 4 DISCUSSION

In this study, we aimed to identify prednisolone responders and non‐responders in patients with AH, estimate the prevalence and influence of ER22/23EK genotype polymorphism, and assess TNF‐α mRNA expression among them. Our data showed significant changes in liver‐function parameters after prednisolone treatment. The non‐responder group was characterized particularly by worsening total bilirubin, higher prothrombin time, higher MDF, and a day‐7 Lille score above the response threshold. We also observed that ER22/23EK genotype polymorphisms of GR were not statistically associated with prednisolone response. Lastly, TNF‐α mRNA was expressed more in AH patients who responded to prednisolone than in those who did not respond to treatment.

The Lille score, which incorporates biochemical and clinical parameters, is a validated measure of response to prednisolone in AH. Patients with Lille score <0.45 are described as responsive, meaning that their liver function is improving following glucocorticoid therapy, whereas non‐responders are typically considered for discontinuation of glucocorticoid treatment at Day 7 (Hakizimana et al., 2024; Popa et al., 2007). In the present cohort, non‐response was not explained by ER22/23EK genotype distribution. Instead, the clinical drivers were consistent with the Lille model itself, particularly bilirubin evolution and coagulation‐related severity. This observation supports the interpretation that clinical disease trajectory remained more predictive than the tested rare GR polymorphism.

As prednisolone is generally considered anti‐inflammatory in severe inflammatory diseases such as AH (Hakizimana, Ntakirutimana, et al., 2026; Hernández Cruz et al., 2008), we tested the hypothesis that prednisolone response would be mediated through downregulation of TNF‐α. We expected TNF‐α mRNA to be higher in non‐responders and lower in responders. However, the observed pattern was the opposite: responders had higher TNF‐α mRNA expression at Day 7 than non‐responders. This finding should not be interpreted as evidence that prednisolone is pro‐inflammatory. Rather, it may reflect temporal immune remodeling, survivor/responder biology, or the fact that TNF‐α alone is insufficient to capture the full steroid‐response network. The lack of IL‐1β, IL‐6, GR, 11β‐hydroxysteroid dehydrogenase (HSD), PCK1, and G6PC expression data prevents a mechanistic conclusion about the transcriptional consequences of prednisolone in this cohort.

Beyond the blood biochemical parameters that have been widely investigated among AH patients following corticosteroid treatments, the key strength of the current study lies in its pharmacogenetic focus. We tested the hypothesis that prednisolone response would be influenced by ER22/23EK (rs6189 and rs6190) genotype. Contrary to our expectation, the genotypes showed no significant association with response to prednisolone treatment under ordinary, dominant, or recessive models. This suggests that the ER22/23EK polymorphism was not a major determinant of prednisolone response in this Indian AH cohort (van Winsen et al., 2007). However, because ER22/23EK is uncommon and may affect GR transactivation more than transrepression, this finding does not exclude the possibility that other NR3C1 polymorphisms, GR isoform expression, 11β‐HSD activity, or liver‐specific glucocorticoid‐responsive genes could influence corticosteroid response in AH.

### 4.1 Limitations

This study has several limitations. First, the sample size was modest, particularly in the non‐responder group, and ER22/23EK is a relatively rare polymorphism; therefore, the study may be underpowered to detect small genotype effects. Second, only TNF‐α mRNA was assessed, while other inflammatory transcripts, including IL‐1β and IL‐6, were not measured. Third, liver‐specific glucocorticoid‐response genes such as PCK1 and G6PC, important enzymes like GR, 11β‐HSD, and GR isoform DNA‐binding activity and transcriptional function, were not assessed. Fourth, PBMC gene expression can serve as an immune proxy but may not fully represent hepatocyte or hepatic immune‐cell responses in AH. Fifth, prednisolone adherence and drinking status were assessed clinically, but quantitative pill‐count and alcohol‐consumption datasets were not available for all patients.

## 5 CONCLUSIONS

In conclusion, TNF‐α mRNA expression was higher at day 7 among prednisolone responders than among non‐responders with AH. More importantly, this study suggests that prednisolone response among AH patients was not statistically associated with ER22/23EK (rs6189 and rs6190) genotype in this cohort. This conclusion should not be interpreted as excluding all possible glucocorticoid‐receptor‐mediated mechanisms of steroid response in AH.

## AUTHOR CONTRIBUTIONS

**Navakanth Raju Ramayanam:** Conceptualization; data curation; formal analysis; investigation; methodology; resources; software; supervision; visualization. **Rajesh Nanda Amarnath:** Conceptualization; data curation; formal analysis; investigation; validation; visualization. **Sarad Pawar Naik buke:** Conceptualization; investigation; methodology; project administration; resources; supervision; visualization. **Vijayakumar Thangavelmahalingam:** Data curation; investigation; methodology; resources; supervision; validation; visualization. **Abdullateef Isiaka Alagbonsi:** Conceptualization; formal analysis; investigation; methodology; visualization. **Hope Onohuean:** Conceptualization; data curation; formal analysis; investigation; methodology; project administration; resources; software; validation; visualization.

## FUNDING INFORMATION

No external grant funding was received. The clinical and laboratory experiments were supported by routine departmental/institutional resources at SRM Medical College Hospital and Research Center.

## CONFLICT OF INTEREST STATEMENT

The authors declare that they have no competing interests.

## ETHICS STATEMENT

The Hospital's Board Ethics of the SRM Medical College Hospital and Research Center, Kanchipuram, approved this research (No. 2363/IEC/2021). The study was conducted in accordance with the ethical principles of the Declaration of Helsinki.

## Data Availability

The de‐identified datasets underlying the biochemical, genotype, and qPCR findings will be made available by the corresponding author upon reasonable request, subject to institutional ethics approval and patient‐confidentiality requirements.
